# Checkpoint Inhibitors and Beyond: A Systematic Review of Immunotherapy in Cutaneous Malignancies

**DOI:** 10.7759/cureus.98959

**Published:** 2025-12-11

**Authors:** Yasir Rashid, Kartika Devi S, Tomas Faustino Gonzalez-Espinosa, Juhi Jain, Mujahed Dalain, Rayyan Baig, Giuseppe Antonio D'Amico, Adetola G Mowo-Wale, Mariia Khomchenko, Nima Baby, Dana Yateem, Axel Duhamel, Ramsha Ali

**Affiliations:** 1 Department of Dermatology, Al-Mustansiriyah University, Baghdad, IRQ; 2 Department of Dermatology, Sri Ramachandra Institute of Higher Education and Research, Chennai, IND; 3 Department of Radiation Oncology, American British Cowdray Medical Center, Mexico City, MEX; 4 Department of Respiratory Medicine, University Hospitals Birmingham NHS Trust, Birmingham, GBR; 5 Faculty of Medicine, University of Latvia, Riga, LVA; 6 Department of Dermatology, Batterjee Medical College, Jeddah, SAU; 7 Department of Plastic and Reconstructive Surgery, Azienda Ospedaliera Universitaria Policlinico "G. Martino", Messina, ITA; 8 Department of Internal Medicine, Obafemi Awolowo College of Health Sciences, Sagamu, NGA; 9 Department of Pulmonology, Charles University, Prague, CZE; 10 Department of Internal Medicine, University Hospitals of Leicester NHS Trust, Leicester, GBR; 11 Department of Medicine, The Shrewsbury and Telford Hospital NHS Trust, Shrewsbury, GBR; 12 Department of Internal Medicine, Università degli Studi di Milano, Milano, ITA; 13 Department of Medicine and Surgery, People's University of Medical and Health Sciences for Women, Nawabshah, PAK

**Keywords:** braf/mek inhibitors, checkpoint inhibitors, cutaneous squamous cell carcinoma (cscc), immunotherapy, : melanoma, merkle cell carcinoma

## Abstract

Skin cancers represent a major health concern, and there is a need for more effective treatment approaches, among which immune checkpoint inhibitors have become a particularly important recent development.

This study aimed to explore the efficacy and tolerability of immune checkpoint inhibitors, intratumoral immunotherapies, targeted agents, and their combinations in advanced cutaneous malignancies.

A Preferred Reporting Items for Systematic Reviews and Meta-Analyses (PRISMA)-conform review of PubMed (2012-2024) identified 26 studies, including randomized trials, observational cohorts, network meta-analyses, and systematic reviews, evaluating checkpoint inhibitors, anti-PD-1/PD-L1and anti-CTLA-4. Outcomes included progression-free survival (PFS), objective response rate (ORR), overall survival (OS), biomarkers, and treatment-related adverse events.

This meta-analysis of 26 studies (2012-2024) evaluated treatments for cutaneous malignancies, including melanoma, basal cell carcinoma (BCC), cutaneous squamous cell carcinoma (cSCC), and Merkel cell carcinoma (MCC), covering systemic immunotherapies (PD-1, CTLA-4), combination checkpoint inhibitors, and novel approaches like IL-12 electroporation. Melanoma: PD-1 therapies showed durable benefits; ipilimumab retreatment yielded 42% two-year survival. MCC: Avelumab achieved a median OS of 12.9 months. cSCC: Nivolumab PFS 8.2 months; cemiplimab 12-month PFS >53%. Targeted therapy: BRAF/MEK inhibitors reached OS ~33 months. Emerging strategies: TIL-based and neoadjuvant immunotherapy showed high pathological and durable responses. Overall, combination therapies consistently outperformed monotherapies in survival and response. Adverse events were common, especially with combination therapy, with severe immune-related toxicities reported in 30-59% of cases, while monotherapies were generally safer. Overall, immunotherapy offers substantial, often long-lasting benefits, though careful patient selection and monitoring are essential to balance efficacy and toxicity.

Combination immunotherapies and targeted regimens are more effective for advanced melanoma, although they have increased toxicity.

## Introduction and background

In spite of representing only 1% of all skin cancers, melanoma is responsible for most skin-cancer-related deaths, and it is the 23rd most common cause of cancer-related death [1,2]. Its incidence has dramatically risen since 1975, reaching over 324000 cases in 2020, and is expected to exceed half a million by 2040 [3]. 

In this context, melanoma treatment has progressed from traditional surgery and late-stage chemotherapy to a broader range of systemic options, including modern immunotherapies and combination approaches. [4]. The immune system is key in controlling melanoma, and checkpoint inhibitors have shown strong potential by boosting immune responses, marking a major breakthrough in cancer therapy.

Skin cancers are generally categorized into two primary types: non-melanoma skin cancers (NMSC), also known as keratinocyte carcinomas (KCS), and melanoma skin cancers (MSC). NMSC includes basal cell carcinoma (BCC) and squamous cell carcinoma (SCC), which account for most of these cases [5]. The primary environmental risk factor for all skin cancer is ultraviolet (UV) radiation [6]. BCC is frequently associated with the abnormal activation of the Hedgehog signaling pathway [7], while SCC typically involves mutations in genes such as TP53, RAS, CDKN2A, and NOTCH [8]. There are also other skin cancers, such as Merkel cell carcinoma (MCC) and Kaposi sarcoma, which, although less frequent, are clinically significant due to their aggressive behavior and strong association with viral infections and immunosuppression [9,10].

Melanoma is aggressive and often metastasizes, commonly driven by NRAS and BRAF-V600E mutations that activate MAPK and PI3K pathways. Tumors evade immunity by reducing antigen expression and recruiting suppressive cells. Immunotherapy - especially checkpoint inhibitors targeting PD-1 and CTLA-4 - has transformed treatment, delivering durable responses and improved survival [11]. Given their high tumor mutational burden (TMB), skin cancers generally respond favorably to immunotherapy, which now complements traditional approaches such as surgery, phototherapy, and topical therapies [12].

Immunotherapy represents a significant advancement in treating skin cancers, primarily driven by the development of immune checkpoint inhibitors (ICIs) targeting PD-1, PD-L1, and CTLA-4 pathways. This systematic review synthesizes current evidence on the clinical efficacy and safety across various skin cancers. The strongest data support their use in advanced melanoma, where these therapies have notably improved overall survival (OS) and produced durable response rates. In NMSCs, such as cutaneous squamous cell carcinoma (cSCC) and MCC, emerging studies report encouraging outcomes, particularly in patients with high TMB or elevated PD-L1 expression [11-13].

The review also highlights increasing interest in combination strategies, including immunotherapy with targeted agents or radiotherapy, to enhance efficacy and overcome resistance. Nonetheless, variability in patient response, limited predictive biomarker reliability, and immune-related adverse events (irAEs) remain significant challenges. While most adverse effects are manageable, they require vigilant monitoring and timely intervention. Overall, these findings highlight the expanding role of immunotherapy in cutaneous oncology. However, further high-quality studies are essential to refine patient selection, establish reliable biomarkers, and optimize therapeutic regimens across the spectrum of skin cancers.

One shortcoming is the clinical validation of biomarkers. Multiple studies highlight current biomarkers, such as PD-L1 and TMB expression; however, none are currently adequate for guiding clinical decisions, such as selecting between immunotherapy and targeted therapy [14-20]. Several studies discuss the efficacy of combination immunotherapy regimens (e.g., nivolumab plus ipilimumab). These regimens are more effective in certain situations but present a higher risk of adverse effects [18,21-23]. Underrepresenting specific population groups (e.g., older patients or those with multiple comorbidities, and ethnically diverse populations) presents a further limitation in reviewed studies [22]. Since treatment responses, levels of toxicity, and disease progression can vary across different groups, population heterogeneity is an essential factor in assessing the real-world effects of immunotherapy in cutaneous cancer treatment. Additionally, there is a lack of data from large-scale comparative studies on immunotherapy for cutaneous skin cancer, particularly regarding the findings of new checkpoint inhibitors and the immune response to neoplasia.

## Review

Methods

Brief Introduction

This review, following Preferred Reporting Items for Systematic Reviews and Meta-Analyses (PRISMA) 2020 guidelines [24], synthesizes recent high-quality evidence on immunotherapy for adult skin cancer patients, with a comprehensive PubMed search conducted on March 17, 2025.

Inclusion and Exclusion Criteria

The inclusion and exclusion criteria are listed in Table 1.

**Table 1:** Inclusion and exclusion criteria.

Criteria	Inclusion	Exclusion
Population	Adults (≥18 years) with skin cancer	Pediatric or pregnant patients
Intervention	Any immunotherapy, including checkpoint inhibitors	Non-immunotherapy interventions
Comparators	Standard treatment, placebo, or no treatment	Studies without comparators irrelevant to the objective
Study Types	RCTs, observational studies, diagnostic accuracy studies, validation studies, meta-analyses, systematic reviews, methodological papers	Animal studies, preclinical experiments, non-English studies
Language	English	Non-English
Publication Date	Last 12 years (2013-2025)	Older than 12 years
Relevance	Focused on immunotherapy in skin cancer	Unrelated topics

Data Sources

Meta-analysis, clinical trials, randomized controlled trials, and systematic reviews from PubMed were used as data sources. Medical Subject Headings (MeSH) terms like “immunotherapy,” “skin neoplasms,” or “cutaneous malignancies” were incorporated to find the relevant articles for the comprehensive search.

Search Strategy

A comprehensive literature search was conducted using PubMed to identify relevant studies. The search was designed to capture peer-reviewed articles published in English that addressed the key concepts of our research focus.

Relevant keywords, MeSH terms, and Boolean operators were used to refine the search. Examples of terms included “(e.g., immunotherapy),” “(e.g., melanoma),” “(e.g., checkpoint inhibitors),” and their combinations such as ((“Immunotherapy”[MeSH Terms] OR “Immunotherapy”[All Fields] OR “immunotherapies”[All Fields] OR “immunotherapy s”[All Fields] OR “Immunotherapy”[MeSH Terms]) AND “Skin Neoplasms”[MeSH Terms]) OR “Cutaneous Malignancies”[All Fields]) AND ((ffrft[Filter]) AND (clinical trial[Filter] OR meta-analysis[Filter] OR randomized controlled trial[Filter] OR systematic review[Filter])).

Selection Process

Title and abstract screening were conducted using the Rayyan AI web-based tool (Rayyan Systems Inc., Cambridge, MA) [25]. Filters were applied to restrict the search to human studies and specific publication years as needed. All titles and abstracts retrieved were screened for relevance. Full texts of potentially eligible studies were reviewed for inclusion based on predefined criteria aligned with this paper's objectives. References of selected articles were also screened to identify additional pertinent studies.

**Figure 1:**
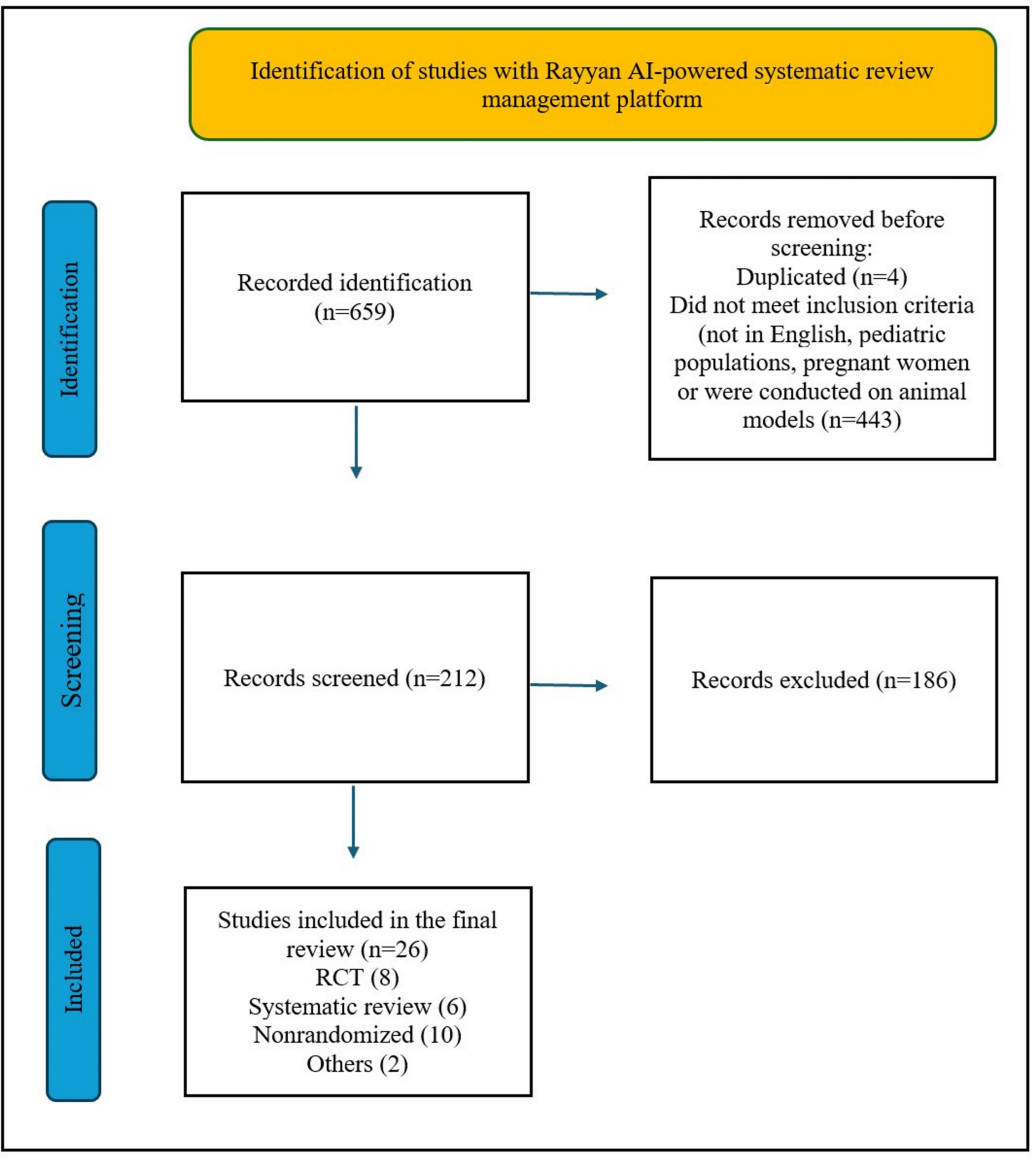
The Preferred Reporting Items for Systematic Reviews and Meta-Analyses (PRISMA) flow diagram outlines the article selection process. These studies were identified with the Rayyan AI-powered systematic review management platform. Rayyan AI for screening [25]

The PRISMA flow diagram (Figure 1) outlines the article selection process.

Risk of Bias Assessment 

To assess the risk of bias in the included studies, we performed a comprehensive bias analysis using RevMan 5.4 (The Cochrane Collaboration, Oxford, UK). This evaluation examined key domains, including selection bias, performance bias, detection bias, attrition bias, and reporting bias. Each study was independently assessed by three reviewers, and discrepancies were resolved through discussion. The results of this analysis provided insight into the methodological quality of the included trials and informed the interpretation of our findings.

Results

This meta-analysis includes 26 randomized controlled trials, observational studies, network meta-analyses, and systematic reviews between 2012 and 2024. These studies evaluated various interventions for skin cancer, such as melanoma, BCC, cSCC, and MCC. The interventions included systemic immunotherapies like anti-PD1 and anti-CTLA4 monotherapy, combination ICIs, targeted therapies like BRAF and MEK inhibitors, and innovative treatments such as intertumoral IL-12 electroporation. Studies were excluded if they were not published in English, involved pediatric populations, included pregnant women, or were conducted on animal models.

Random Sequence Generation

Random sequence generation ensures unbiased assignment of participants to treatment groups, often using computer-generated random numbers or block randomization methods. The KEYNOTE-716 trial [22], CheckMate 141 [23], and the nivolumab vs. dacarbazine trial likely utilized robust randomization techniques. However, the available data does not specify the procedures used (e.g., stratified or block randomization). The inclusion of the “Cochrane Risk of Bias Tool” in João Lima’s 2017 meta-analysis indicates a commitment to standards for evaluating randomization quality [14,15]. In contrast, retrospective or non-randomized studies, such as Jason Luke’s 2019 real-world analysis, lack this protection, revealing inconsistencies in bias control throughout the dataset [17]. Where randomization methods were not reported, risk was classified as “unclear” according to Cochrane criteria.

Blinding of Outcome Assessment (Detection Bias)

Blinding of outcome assessment varied notably across studies. Open-label and retrospective trials had high detection bias risk [17,26], while systematic reviews and meta-analyses showed unclear risk due to inconsistent reporting [14,27,28]. Some randomized trials lacked blinding details [2,20], whereas double-masked phase 3 trials presented low risk [18]. Overall, inconsistent blinding and reporting practices highlight a need for greater transparency in trial design.

Incomplete Outcome Data (Attrition Bias)

While most studies did not explicitly mention attrition bias, some acknowledged issues such as missing data, loss to follow-up, or small sample sizes. For example, studies on avelumab [26] and prolgolimab [29] reported missing biomarker or long-term survival data, while others, like adjuvant pembrolizumab [22], had limited follow-up compliance. Smaller trials, such as the NIVO-TIL study, faced sample size and long-term tracking challenges [19]. Conversely, larger RCTs like nivolumab vs. dacarbazine and meta-analyses using Cochrane standards demonstrated a lower attrition risk due to robust methodologies [18,28]. Studies employed strategies like intention-to-treat analysis and sensitivity analyses to address these biases. The conclusion emphasizes the need for better follow-up protocols, transparent reporting, and ITT analysis to minimize attrition-related biases in future research. 

Most studies did not explicitly report attrition bias, though some noted missing data, loss to follow-up, or small sample sizes. Trials, such as those by Kaufman et al. [26] and Tjulandin et al. [29], lacked complete biomarker or survival data, while Khattak et al. [22] faced limited follow-up. Smaller studies (e.g., NIVO-TIL) struggled with sample size and tracking, whereas larger RCTs and Cochrane-based meta-analyses showed lower attrition risk through stronger methods. Use of ITT and sensitivity analyses helped mitigate bias, underscoring the need for improved follow-up and transparent reporting in future studies.

Allocation Concealment

In this review, studies, such as those by Ascierto et al. [18], Kaufman et al. [26], and Olson et al. [30] were identified as having a low risk of bias due to their articulated and robust concealment methods [18,26,30]. In contrast, a high risk of bias was observed in various single-arm or non-randomized trials, including those of L’Orphelin et al., Tjulandin et al., Migden et al., Hasmat et al., Ríos-Viñuela et al., and Rischin et al., where allocation procedures were inherently unblinded or poorly described [19,29,31-34]. Additionally, some studies did not provide sufficient methodological detail, resulting in an unclear risk assessment. The lack of proper allocation concealment can lead to potential biases in treatment assignment, whether deliberate or accidental, which can compromise baseline comparability between groups and undermine the overall generalizability of the results.

Blinding of Participants and Personnel (Performance Bias) 

This poses a significant challenge in open-label trials and immunotherapy research, as demonstrated by Kaufman et al., Greaney et al., and Lövgren et al., who showed a high risk of bias [26,35,36]. Only a select number of studies, such as those conducted by Boutros et al., have successfully employed blinding [14]. The absence of blinding can influence participants’ responses and behaviors, likely exaggerating perceived treatment benefits due to placebo effects or inconsistent care, thereby jeopardizing the internal validity of trial results.

Reporting Bias

Studies by Boutros et al., Jamal et al., and Olivier et al. showed minimal selective reporting risk due to adherence to prespecified protocols [14,16,21]. In contrast, early-phase and observational studies had a higher risk of post hoc changes or omitted results [35,36]. Such bias may obscure safety signals and overstate efficacy, reducing research reliability and reproducibility.

**Figure 2:**
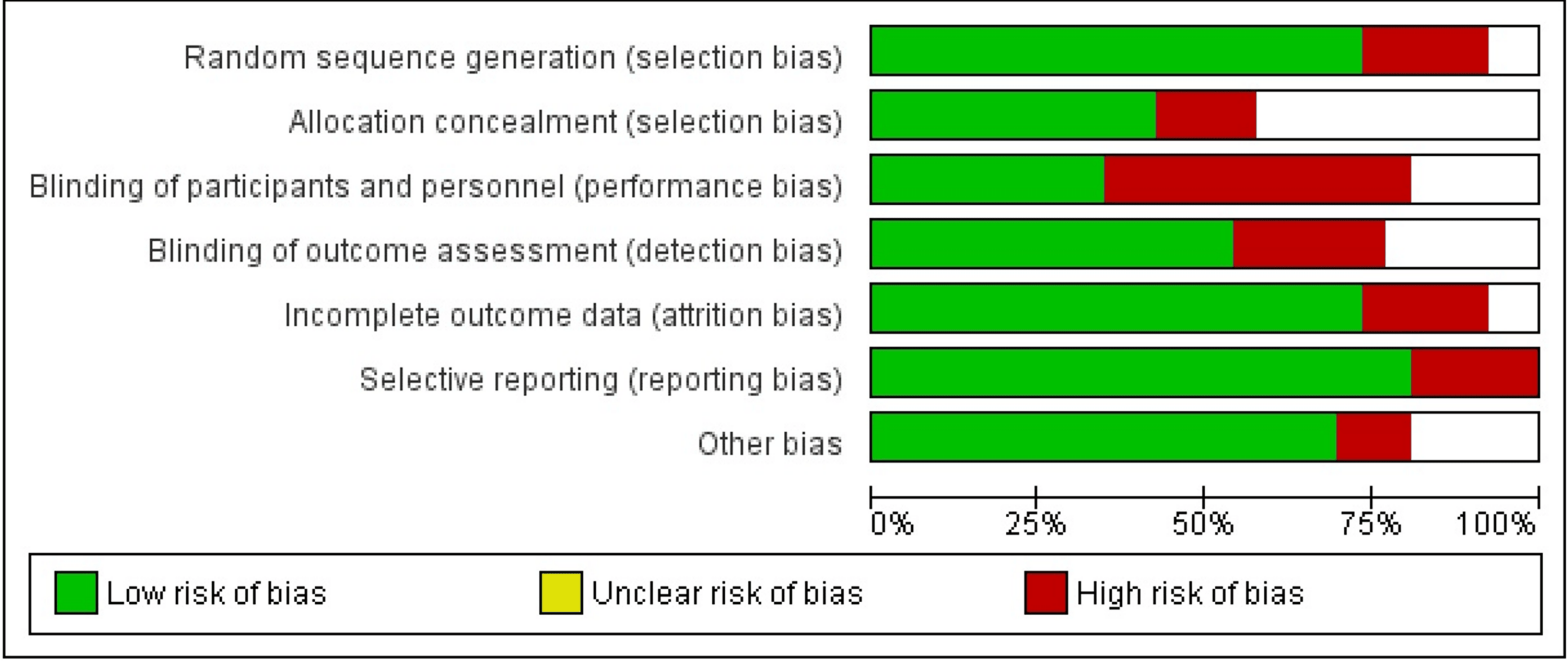
Overall summary of risk of bias domains evaluated using the Cochrane Risk of Bias Tool. Each domain is represented as a horizontal bar indicating the percentage of studies assessed as having low risk (green), unclear risk (yellow), or high risk (red) of bias across seven domains: random sequence generation, allocation concealment, blinding of participants and personnel, blinding of outcome assessment, incomplete outcome data, selective reporting, and other biases.

**Figure 3:**
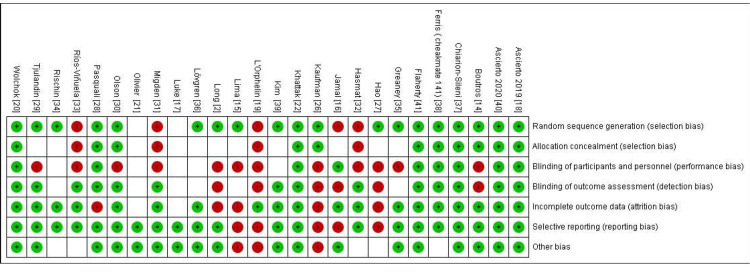
Risk of bias assessment by individual study across the seven standard domains. Each cell represents the judgment for a specific domain in a given study: green (+) indicates low risk, red (-) indicates high risk, and white (blank) indicates unclear or not reported. The studies are listed by first author, publication year, and reference for citation in square brackets.

Risk of bias was evaluated using the Cochrane Risk of Bias Tool. Figure 2 summarizes the proportion of studies judged to have low, high, or unclear risk across different domains. The domain-specific assessment by the study is shown in Figure 3, with high risk most frequently observed in blinding of outcome assessment, incomplete outcome data, and selective reporting.

Efficacy outcomes and survival rates

Melanoma Outcomes

The primary outcomes reported in the studies centered on OS and progression-free survival (PFS), which are vital measures of treatment effectiveness in skin cancer. Dual checkpoint inhibition consistently demonstrated better survival results than monotherapy or standard chemotherapy among the various immunotherapy approaches. For example, in the landmark CheckMate 067 trial, the combination of nivolumab and ipilimumab resulted in a median OS of 72.1 months, significantly better than nivolumab alone (36.9 months) or ipilimumab alone (19.9 months). This indicates that dual checkpoint inhibition is more effective for advanced melanoma [20].

Further evidence supporting these findings came from meta-analyses by da Silveira Nogueira Lima et al. and Pasquali et al. [15,28]. They confirmed that BRAF, MEK, and PD-1 inhibitors help extend OS and PFS. Anti-PD-1 monotherapy proved to be the best choice among these therapies because it is highly effective and easier to tolerate than combination therapies.

In the MIRACULUM phase II trial, which tested prolgolimab, a new anti-PD-1 monoclonal antibody, patients with advanced melanoma received either 1 mg/kg biweekly (Q2W) or 3 mg/kg every three weeks (Q3W). The study reported that the two-year OS rates were 57.1% for the Q2W group and 46.0% for the Q3W group, demonstrating significant and lasting survival benefits for both treatment schedules [29].

The possibility of retreatment with ICIs was investigated by Chiarion-Sileni et al. [37]. They studied 51 patients with advanced melanoma who had previously benefited from ipilimumab. After their disease progressed, they were retreated with ipilimumab (3 mg/kg every three weeks), resulting in a two-year survival rate of 42% and a median post-retreatment OS of 21 months. Notably, 55% of the patients regained disease control, which included complete or partial responses or stable disease. These results suggest that reevaluating ipilimumab could be beneficial for some patients.

In a real-world retrospective study, Luke et al. found that in advanced melanoma, targeted therapy (BRAF/MEK inhibitors) achieved a median OS of 33.1 months, while OS was not reached for immunotherapy (nivolumab or pembrolizumab), indicating prolonged survival [17]. Immunotherapy patients had shorter treatment durations but longer treatment-free intervals, suggesting better long-term disease control.

Hao et al. meta-analyzed six RCTs (3,284 advanced melanoma patients) and found that anti-PD-1 monotherapy and nivolumab/ipilimumab combination significantly improved OS and PFS versus chemotherapy or ipilimumab alone. OS hazard ratios ranged from 0.63 to 0.73 for monotherapy and 0.55 for combination, showing up to 45% reduced death risk. PD-1 therapies demonstrated durable responses, supporting their use as first-line treatment, with combination therapy reserved for cases where benefits outweigh added toxicity [27].

The NeoTrio trial [2] reported an 80% pathological response in resectable stage III melanoma with concurrent neoadjuvant immunotherapy, outperforming pembrolizumab alone or sequential therapy. Similarly, the NIVO-TIL trial showed durable responses of nine months to 3.4 years with sequential anti-PD-1 therapy followed by TIL infusion, highlighting strong effectiveness beyond survival metrics [19].

Merkel Cell Carcinoma

In the MCC study, Kaufman et al. assessed avelumab among chemotherapy-refractory metastatic cases. The trial revealed a median OS of 12.9 months, with one-year and two-year OS rates of 52% and 39%, respectively. These findings are particularly significant given the historically limited treatment options available and the aggressive nature of MCC [26].

Non-melanoma Skin Cancers

In metastatic cSCC, Ferris et al. (CheckMate 141) reported a median PFS of 8.2 months with nivolumab in patients without prior cetuximab exposure [38]. Migden et al. observed a 12-month PFS rate of over 53% with cemiplimab [31], while Ríos-Viñuela et al. reported a median PFS of 5.9 months (range 1.9-15.5) [33]. The longest PFS was seen in Richen et al., reaching 18.4 months [34].

Response rates

Studies on immunotherapy for skin cancer consistently showed clinical benefits in response rates, PFS, and duration of response (DOR). L’Orphelin et al. reported 75% of patients responding to anti-PD-1 followed by TIL infusion, with responses lasting nine months to over three years and immune activity persisting beyond two years [19]. Lövgren et al. observed strong immune activation and durable T-cell memory with TIL plus dendritic cell vaccines, though one patient experienced IL-2-related toxicity [36].

Encouraging results were also observed in traditional checkpoint inhibitor trials. A study by Ascierto et al. 2019 showed a remarkable 40% response rate and a median PFS of 5.1 months with nivolumab, which was better than conventional chemotherapy [18]. Moreover, Kaufman et al. reported that nivolumab resulted in a 33% response rate among patients with metastatic MCC, and complete responses were observed in 11% of treated individuals [26].

Combination therapies showed superior outcomes: CheckMate 067 reported a median PFS of 11.5 months and 52% five-year OS with nivolumab plus ipilimumab [20], while the NeoTrio trial achieved 70% radiologic and 80% pathological response rates with concurrent neoadjuvant immunotherapy, outperforming sequential or single-agent approaches [2].

Meta-analyses by da Silveira Nogueira Lima et al. and Pasquali et al. confirmed PD-1 inhibitors’ superiority but lacked response rate details, limiting cross-trial comparisons [15,28]. Real-world studies by Luke et al. and Greaney et al. highlighted survival benefits and longer treatment-free intervals linked to predictive biomarkers [17,35].

Despite some inconsistencies and limitations in the reported information, the evidence strongly suggests that immunotherapy offers substantial, often long-lasting advantages, particularly when combined or customized for specific clinical settings.

Special population

A meta-analysis on melanoma brain metastases found that combining ICIs (e.g., nivolumab + ipilimumab) or ICIs with radiotherapy achieved better intracranial control and OS than ICI monotherapy, showing the most favorable survival trends [39]. 

Adverse effects

Immunotherapy has dramatically improved the management of melanoma, MCC, and cSCC; however, it can also trigger adverse effects, particularly those related to the immune system. The irAEs vary across studies, influenced by the specific medication used and whether it is administered alone or in combination with other treatments.

Combination therapies, particularly those that include both nivolumab and ipilimumab, often lead to more severe side effects. In the CheckMate 067 study led by Wolchok et al. [20], nearly 59% of patients receiving nivolumab plus ipilimumab experienced Grade 3 or 4 treatment-related adverse events, including colitis, hepatitis, and thyroid dysfunction. Similar results were observed in the NeoTrio trial, where 30% of participants reported significant side effects, including elevated liver enzymes and fatigue [2].

In contrast, patients who received monotherapy treatments, such as nivolumab or avelumab alone, generally experienced fewer side effects. According to Ascierto et al., only 11% of patients on nivolumab faced serious side effects, compared to 17% in the chemotherapy group [18]. Kaufman et al. found that avelumab resulted in fewer serious complications (5.8%), with most being mild reactions like fatigue or infusion-related symptoms [26].

In patients who received cemiplimab, the most common side effect was G1 grade (fatigue and diarrhea) [31-34,38], and it is noted that it is less severe than nivolumab CheckMate 141 [38].

Some studies have shown that higher doses increase the risk of side effects. For instance, Ascierto et al. noted that administering ipilimumab at 10 mg/kg led to a higher incidence of side effects than a lower dose of 3 mg/kg [40]. Meanwhile, Tjulandin et al. found that the newer medication prolgolimab came with a 12.2% rate of serious adverse effects, which is comparable to other PD-1 inhibitors [29].

Several studies targeted specific patient groups. Kim et al. reported seizures and brain swelling in brain metastasis patients receiving combination therapy [39]. L’Orphelin et al. and Lövgren et al. evaluated TIL-based therapies for advanced melanoma, showing durable responses, though one IL-2-related death occurred in Lövgren et al.’s study [19,36].

Some studies, such as those by Greaney et al. [35] and the meta-analyses by da Silveira Nogueira Lima et al. [15] and Pasquali et al. [28], lacked complete safety data. Overall, while immunotherapy is effective, combination regimens demand close monitoring, as balancing efficacy with manageable toxicity remains crucial.

Discussion

This systematic review provides a wealth of information on the immunotherapy management of skin cancer. However, the main challenge lies in the lack of direct comparisons among the many available treatment options, particularly between immunotherapy as a monotherapy and combination therapy. Despite this limitation, the findings suggest that combination therapy shows a favorable efficacy and safety profile in metastatic cutaneous skin cancers.

This review also highlighted the effectiveness of immunotherapies. It targeted therapies in skin cancers, as reported in twenty-one clinical trials, meta-analyses, and other real-world studies. 

Survival rates and response rates

Table 2 summarizes the key characteristics and outcomes of the studies reviewed.

**Table 2:** Summary of patient characteristics, treatment regimens, outcomes, and adverse events across studies on immunotherapy in skin cancer.

Author	Study design	Sample size	Gender	Biomarker	Population	Primary outcome	Secondary outcome	Results	Adverse events	ECOG status
L'Orphelin et al. [19]	Exploratory, prospective, single-center, open-label, non-randomized, uncontrolled phase I/II trial.	10 previously untreated patients with advanced melanoma.	7 male 3 female	Not given	Patients with metastatic melanoma undergoing anti-PD-1 therapy	The primary objective was to evaluate the clinical and biological safety of adoptive T-cell transfer combined with anti-PD-1 therapy.	The secondary objectives were to evaluate treatment efficacy, duration of clinical response, progression-free survival (PFS), and OS.	Of the four patients treated with autologous TILs plus nivolumab, three (75%) achieved objective responses - two CRs and one PR. Prior to TILs, one had PR and two had SD on nivolumab alone, highlighting the added benefit of TILs. Responses lasted 9 months to 3.4 years.	Five SAEs occurred, four of which were unrelated to TIL/IL-2/anti-PD-1 therapy: myocardial infarction (MI) (only SAE considered treatment-related) - possibly linked to nivolumab (anti-PD-1) due to pre-existing cardiovascular risks.	Not given
Jamal et al. [16]	Phase II, open-label, clinical trial	30 patient	8 females 22 m ales	BRAF mutated (V600E) 9 out of 30 Wild BRAF mutation (21 out of 30)	Patients with untreated unresectable stage III or stage IV melanoma	The primary objective was to determine the safety and tolerability of two schedules of ipilimumab in combination with CP	Secondary objectives were to determine putative early cellular and/or molecular biomarkers for therapy response; to measure anti-tumor efficacy (OS, overall response rate (ORR), PFS, and clinical benefit rate (CBR; ORR + stable disease (SD) ≥ 24 weeks)) by irRC and mWHO response criteria	Dual checkpoint blockade with nivolumab and ipilimumab resulted in a median overall survival of 72.1 months, compared to 36.9 months with nivolumab alone and 19.9 months with ipilimumab alone	Grades 3-4 AEs related to ipilimumab were found in 13% of patients had colitis, diarrhea, and endocrinopathies	0 or 1
Ascierto et al. [18]	Randomized phase 3 trial analyzed 3-year overall survival	Nivolumab group: approximately 210 patient. Dacarbazine group: approximately 208 patients. Totaling around 418 participants.	In the nivolumab group,(121 of 210) male; in the dacarbazine group,(125 of 208) male	All patients had confirmed BRAF wild‐type tumors	Patients with unresectable, previously untreated stage III or IV melanoma	Overall survival. Measured at 1, 2, and 3 years At 3 years: Nivolumab: 51.2% overall survival (95% CI, 44.1%–57.9%) Dacarbazine: 21.6% overall survival (95% CI, 16.1%–27.6%) Median OS: Nivolumab: 37.5 months (95% CI, 25.5 months–not reached) Dacarbazine: 11.2 months (95% CI, 9.6–13.0 months) Hazard Ratio for death with nivolumab: 0.46 (95% CI, 0.36–0.59; P < .001)	Response Rates Nivolumab Group: Complete Response (CR): 19.0% (40 of 210 patients) Partial Response (PR): 23.8% (50 of 210	Nivolumab significantly improved overall survival compared with dacarbazine The 3-year OS rate was more than double with nivolumab (51.2% vs 21.6%), with a median OS of 37.5 months versus 11.2 months. Objective response rates (complete and partial) were also higher in the nivolumab arm.	Not given	Not given
Kaufman et al. [26]	Multicenter, single-group, open-label, Phase 2 trial	88 patients with stage IV metastatic Merkel cell carcinoma (mMCC) whose disease had progressed after at least one prior line of chemotherapy	65 male 23 female	-	All patients had stage IV (M1) MCC with distant metastases	The primary outcome of the study was the confirmed objective response rate (ORR), representing the proportion of patients achieving either a complete or partial response to avelumab treatment.	Secondary outcomes included a median DOR not reached (range: 2.8–23.3+ months), median PFS of 2.7 months (95% CI: 1.4–6.9), and median OS of 12.9 months (95% CI: 7.5–not estimable), with 1- and 2-year OS rates of 52% and 39%. Treatment-related adverse events occurred in 62% of patients, with 10% experiencing grade ≥3 events and no treatment-related deaths	This Phase 2 trial evaluated avelumab (10 mg/kg every 2 weeks) in stage IV metastatic Merkel cell carcinoma post-chemotherapy, showing a 31.8% objective response rate (9.1% CR, 22.7% PR). Most responses were durable. Grade 3 treatment-related events occurred in 5% of patients, with no grade 4 events or deaths. Avelumab demonstrated durable efficacy and manageable safety in advanced MCC.	Adverse events occurred in 5% of patients, including lymphopenia (2), increased creatine phosphokinase (1), aminotransferases (1), and cholesterol , with no Grade 4 events or deaths. Serious treatment-related events occurred in 6%, including enterocolitis, infusion reactions, elevated aminotransferases, chondrocalcinosis, synovitis, and interstitial nephritis.	0 or 1
Ascierto et al. [40]	Randomized, multicenter, double-blind, Phase III trial	831	Not given	LDH; wild- type or mutant BRAF tumors	Patients with untreated or previously treated unresectable stage III or IV melanoma	After 61 months, median OS was 15.7 months (10 mg/kg) and 11.5 months (3 mg/kg); in patients with asymptomatic brain metastases, OS was 7.0 and 5.7 months, respectively. For wild-type vs. BRAF-mutant tumors, median OS was 13.8 vs. 33.2 months (10 mg/kg) and 11.2 vs. 19.7 months (3 mg/kg).	Secondary end points included the yearly assessment of OS for up to 5 years, OS based on brain metastases, objective response, progression-free survival and safety	In advanced melanoma, ipilimumab 10 mg/kg improved median OS versus 3 mg/kg (15.7 vs. 11.5 months; HR 0.84, p = 0.04) and 5-year survival (25% vs. 19%), with benefits consistent across subgroups, including brain metastases and BRAF-mutant tumors.	Incidence of grade 3/4 treatment-related AEs was 36% in the 10 mg/kg group vs 20% in the 3 mg/kg group, and deaths due to treatment-related AEs occurred in four (1%) and two patients (1%).	0 or 1
Wolchok et al. [20]	Prospective study	345	Not explicitly reported; presumed balanced due to randomization and large sample size.	BRAF V600 mutation	Adults with advanced melanoma (stage 3 or 4)	Progression - free survival and Overall survival (OS) with nivolumab plus ipilimumab or nivolumab versus ipilimumab	Secondary end points included objective response rate, descriptive efficacy assessments of nivolumab plus ipilimumab versus nivolumab alone, and safety assessment	Median OS: Nivolumab + ipilimumab: 72.1 months Nivolumab alone: 36.9 months Ipilimumab alone: 19.9 months	Not specified	0 or 1
Long et al. [2]	Non-comparative, randomized trial	60	Not explicitly mentioned; assumed balanced across arms	All patients had confirmed BRAFV600 mutation	Patients with resectable stage 3 melanoma	The pathological response rate was 55% with pembrolizumab, 50% with sequential therapy and 80% with concurrent therapy	Objective radiological response ( secondary objective ) was observed in 30% of patients treated with pembrolizumab, 50% of patients treated with sequential treatment and 70% of patients treated with concurrent treatment	In the NeoTrio trial, pathological responses occurred in 11/20 (pembrolizumab), 10/20 (sequential), and 16/20 (concurrent), with complete responses in 6, 3, and 10 patients, respectively. Radiographic responses were 6/20, 10/20, and 14/20 across the same groups.	Treatment-related adverse events affected 75-100% of patients during neoadjuvant treatment 55% of patients in the concurrent therapy arm experienced grade 3/4 adverse events	Not specified
da Silveira Nogueira Lima et al. [15]	Systematic review and network meta-analysis	16 trials on 8 types of therapy, comprising 6849 patients	Gender distribution not individually reported for pooled RCT	BRAF mutation and PD - L1 expression	Patients with metastatic or advanced melanoma	PD-L1 expression and BRAF mutational status as biomarkers of response to immunotherapy	Not given	BRAF-MEK and PD-1 inhibitors had similar OS, both outperforming other treatments. BRAF-MEK showed superior PFS and RR, including vs. CTLA-4-PD-1. PD-L1 expression was not predictive. Best options: BRAF-MEK for BRAF-mutated, PD-1 inhibitors for BRAF wild-type. CTLA-4-PD-1 improved PFS/RR, but OS data are lacking.	Anti-PD1 monoclonal antibodies may result in less toxicity than chemotherapy (RR 0.55, 95% CI 0.31 to 0.97)	Not specified
Pasquali et al. [28]	Network meta-analysis	122 randomized control studies - 28561 participants	No specific details about gender distribution across trials	BRAF - mutant and wild type	Adults diagnosed with metastatic cutaneous melanoma (AJCC TNM stage IV)	Progression-Free Survival (PFS): Immunotherapy (especially PD-1 inhibitors) and targeted therapies improved PFS compared to chemotherapy Because of differences across the studies, a combined estimate could not be calculated	Tumor response and toxicity were assessed using risk ratios (RR) comparing experimental and comparator arms, with RR >1 or <1 indicating favorable or unfavorable effects, respectively, reported with 95% CIs. Quality of life data were reported descriptively due to heterogeneity, and economic analysis used cost-utility based on quality-adjusted life years.	Efficacy: Immunotherapy and targeted therapy offer better disease control than chemotherapy in terms of PFS. Safety: PD-1 inhibitors (e.g., nivolumab, pembrolizumab) had better tolerability and lower high-grade toxicity compared to combination regimens or CTLA-4 inhibitors	33% of patients experienced grade 3/4 adverse events; most common were diarrhea, hepatitis, and hypophysitis	Not specified
Tjulandin et al. [29]	Multicenter open-label parallel-arm phase II trial randomized	126 patients	45% male 55% female	BRAFV600E/K mutation status and PD-L1 expression	Adult patients, with stage II-IV unresectable or metastatic melanoma,, two arms prolgolimab 1 and 3 mg/kg	ORR assessed irRECIST by independent central review	PFS, OS, DCR, TTR and DOR.	ORR 38.1% arm 1, 28.6 arm 2, Two year PFS 33.3% arm 1 and 30.2% in arm 2, two year OS 57.1% arm 1, 46% arm 2	Grades III-IV treatment-related adverse events occurred 12.7%, in arm 1, and 3.2 % in arm 2	ECOG 0 and 1 (55% and 45%)
Hao et al. [27]	Systematic review and meta-analysis of RCTs	11 reports from 6 RCTs, 3284 patients with metastatic melanoma	60% male	BRAF - mutant and wild type, PD-L1 expression, TMB, inflammatory gene expression signature	Patients with metastatic melanoma 3 subgroups: nivolumab/pembrolizumab vs. chemotherapy, nivolumab vs. ipilimumab and nivolumab/ipilimumab vs. ipilimuma	PFS and OS in 4 trials and ORR in 2 trials	Toxicity estimated by grade 3 and grade 4 adverse events	Anti-PD1 monotherapy and nivolumab plus ipilimumab, improved ORR and prolonged PFS of patients with advanced melanoma	Less risk of adverse events in the anti-PD1 treatment group vs chemotherapy and ipilimumab group	0 or 1
Kim et al. [39]	Systematic review and meta-analysis	1,366 patients 11 studies, with 14 cohorts (3 ICI combination, 5 with ICI combined with radiotherapy and 6 with ICI monotherapy)	60% male	PD-L1 expression, BRAF mutation status	Patients with malignant melanoma brain metastasis	Intracranial ORR and/or DCR	Safety associated outcomes	ICI combination therapy or ICI combined with radiotherapy showed better local efficacy than ICI monotherapy for treating melanoma brain metastasis.	Grades 3 or 4 adverse events rate, was significantly higher with ICI combination therapy. CNS related adverse events were similar	0 or 1
Luke et al. [17]	Retrospective, observational	440	168 (59.4%) male	Not given	Confirmed BRAF V600 activating mutation	The primary study cohorts for analysis were patient treated in 1st Line with any combination targeted therapy (dabrafenib + trametinib or vemurafenib + cobimetinib ) versus any 1st line Immunotherapy nivolumab , pembrolizumab, or the combination of ipilimumab plus nivo (ipi/nivo)	Not given	Treatment duration and outcomes: Time on treatment was longest for TT (11.4 month) vs. IO (7.2 month), shortest for ipi/nivo (4.6 month); time off treatment was longer for IO (1.5 month) vs. TT (0.6 month). OS was similar between TT (33.1 month) and IO (not reached). Post D+T, most 2nd-line treatments were PD-1 monotherapy (59.8%); post ipi/nivo, D+T was most common (62%). ORR was higher for TT than IO: provider-reported 79.2% vs. 71.3%, RECIST 60.1% vs. 45.9%; D+T outperformed PD-1 monotherapy (60.6% vs. 42.3%).	Not given	ECOG-PS (0/1 or ≥2)
Greaney et al. [35]	Non randomized prospective observational study with retrospective analysis	29 patients with cancer who received immune checkpoint blockade (ICB) therapy		PD-1; Ki67; IFNy; CD3; CD8; TCR clonality	Non-small cell lung cancer (NSCLC) and melanoma	Determine whether the presence of CD39⁺ CD8 T cells in peripheral blood could serve as a predictive biomarker for clinical response to immune checkpoint blockade (ICB) therapy. The researchers aimed to establish whether a higher baseline level of these cells correlated with better treatment outcomes	Investigation into the characteristics of CD39⁺ CD8 T cells, particularly their tumor specificity, phenotypic features, and their association with progression-free survival (PFS) and overall survival (OS). The study assessed whether these cells exhibited markers of T cell exhaustion, clonal expansion, and tumor reactivity, all of which would suggest their active role in the anti-tumor immune response	Patients responding to ICB therapy had higher baseline CD39⁺ CD8⁺ T cells (21.3% vs. 11.4%)	Not given	Not given
Olivier et al. [21]	Phase III, randomized, open-label clinical	945	Not given	PD-L1; TMB; MSI	Patients with untreated unresectable stage III or stage IV melanoma	CheckMate 067 primarily assessed overall survival, comparing nivolumab, ipilimumab, and their combination in untreated, unresectable stage III/IV melanoma.	The secondary outcomes included progression-free survival (PFS), the proportion of patients who experienced a complete or partial response, and quality of life (QoL) measures, as well as the incidence of treatment-related adverse events (AEs)	In CheckMate 067, nivolumab + ipilimumab showed the longest median OS (72.1 months) vs. nivolumab alone (36.9 months) and superior PFS (11.5 vs. 6.9 months), but had higher grade 3/4 toxicity (59% vs. 24-28%) and more early discontinuations. Treatment-free interval was longest with the combination (27.6 months), though toxicity raised quality-of-life concerns.	Not given	Not given
Chiarion-Sileni et al. [37]	Clinical trial	855h	Male (24), Female (27)	Unspecified	Patients with life-threatening unresectable stage III or stage IV melanoma were eligible to be included in the EAP if they had failed to respond or were intolerant to at least one systemic therapy and if no alternative treatment option was available.	126 patients had disease progression following a response to ipilimumab induction therapy	51 patients (6%) were retreated with ipilimumab 3 mg kg^−1^. Of these retreated patients, 31 patients had irSD lasting 3 months as their best response to induction therapy, and 20 patients had an irPR with induction therapy.	Of 51 patients retreated with ipilimumab, 55% regained disease control and 42% were alive at 2 years; median OS was 21 months. Treatment-related AEs occurred in 22%, were mostly mild-to-moderate, resolved in 4 days, with no new toxicities.	Among 51 patients retreated with ipilimumab 3 mg/kg, 39% experienced drug-related AEs during induction (2 grade 3). Upon retreatment, 27% had any AE, 22% were drug-related; 16% had grade 1-2 and 6% had grade 3-4 treatment-related AEs.	ECOG status: 0 in 36 patients (71%), 1 in 15 (29%); time from diagnosis median 50 months (range 4-199).
Lövgren et al. [36]	Single-center, open-label, Phase I clinical trial	14	Not given	PBMCs, checkpoint marker PD-1, TILs, antigen responses, cytokine production.	Progressive inoperable stage III or stage IV (according to AJCC) malignant melanoma	PFS was 3 months, >18 months , and >42 months); not reported for others. OS ranged 0.2–>42 months, with median ~7–8 months among reported patients and some achieving long-term survival.	Cohort 1 (safety/optimization) showed only mixed responses or stable disease, not durable. Cohort 2 (combinatorial therapy) had four evaluable patients; all achieved objective responses: two durable CRs (>36 and >18 months), one durable high-quality PR (>42 months), and one short-term PR (<4 months, mixed response by irRC). PET/CT confirmed reduced metabolic activity in responding tumors.	Combination of TIL ACT and tumor lysate-loaded DC vaccination is feasible and safe and we observed impressive clinical responses in all patients treated with the combination.	Severe (Grade 3-5) AEs included febrile neutropenia, neutropenia, and thrombocytopenia (4 patients each), plus hyponatremia, nausea, vomiting, fever, capillary leak, hypoalbuminemia, vasovagal reaction, and one Grade 5 respiratory failure (Patient 14). Patient 10 had no severe AEs.	0-2
Khattak et al. [22]	Randomized, double-blind, placebo-controlled Phase 3 clinical trial	696	Not given	Not given	Histologically confirmed stage IIB or IIC cutaneous melanoma	No significant HRQoL decline occurred with pembrolizumab versus placebo, supporting its tolerability in high-risk stage II melanoma.	The study found no clinically meaningful differences in secondary HRQoL outcomes between treatment arms, reinforcing that pembrolizumab maintains quality of life while providing clinical benefit. Subgroup analyses supported consistent results across patient populations.	The study demonstrates that the significant recurrence-free survival benefit of pembrolizumab in this population comes without compromising patients' quality of life, supporting its favorable benefit-risk profile for adjuvant treatment of high-risk stage II melanoma. These findings complement the primary efficacy results showing pembrolizumab's clinical benefit in this early-stage melanoma population.	Treatment-related adverse events (AEs) occurred in 80% of patients in the pembrolizumab arm versus 60.9% in the placebo arm, and immune-mediated AEs and infusion reactions occurred in 36.2% and 8.4% of patients, respectively.	Not given
Flaherty et al. [41]	Meta-analysis of randomized controlled trials (RCTs)	4416 Review each of the 11 original RCTs included in the analysis	Not given	Not given	We included RCTs in unresectable or metastatic melanoma with dacarbazine as control and any systemic therapy as experimental, reporting HRs for OS and PFS (HR <1 favors experimental; HR >1 favors control).	This meta-analysis found moderate correlation between PFS and OS in metastatic melanoma (R² = 0.46-0.73), stronger for targeted therapies, suggesting PFS can be a reasonable but imperfect OS surrogate. Response rate showed weak correlation with OS (R² = 0.09-0.25), indicating it is unreliable as a survival surrogate.	The study found stronger PFS-OS correlation for targeted therapies than chemotherapy, but weaker association at the individual patient level. Post-progression therapies, including treatment crossover, further reduce PFS-OS correlation, limiting PFS as a surrogate for OS in metastatic melanoma trials.	The study showed moderate PFS-OS correlation (R² = 0.46-0.73), stronger for targeted therapies than chemotherapy, while RR had weak correlation (R² = 0.09-0.25). Patient-level associations were weaker, and post-progression therapies reduced PFS-OS correlation. PFS may be a meaningful but imperfect OS surrogate; RR is unreliable.	Not given	Not given
Olson et al. [30]	Prospective clinical trial; in this open-label, single-arm phase II trial	70 patient	47 male, 23 female	Patients had BRAFV600 mutations	Prior treatments included 60 on anti-PD-1 antibody alone and 10 on anti-PD-1/L1 antibody-based combinations.	To assess the efficacy of pembrolizumab plus low-dose ipilimumab in advanced melanoma in patients refractory to an antiprogrammed cell death protein-1 (PD-1) and programmed death ligand-1 (PD-L1) antibody.	Secondary objectives included summarizing PFS (defined as time on study treatment until immune- related progressive disease, clear clinical progression, or death) and safety.	The median progression-free survival was 5.0 months, and the median overall survival was 24.7 months. The median duration of response was 16.6 months. There was no difference in median time on prior anti-PD1/L1 or time to PD1 + CTLA4 initiation between responders and nonresponders	The most common adverse events (AEs) included fatigue (48%), pruritus (42%), rash (36%), diarrhea (33%), and arthralgia (30%). Grades 3-4 immune-related AEs occurred in 39% of patients, with colitis (12%), hepatitis (9%), and hypophysitis (6%) being the most frequent severe toxicities.	Not given
Boutros et al. [14]	A network meta-analysis	9070 metastatic melanoma patients treated in 18 randomized clinical trials were included in the network meta-analysis.	Not given	Not given	Metastatic melanoma patients	RCTs in untreated advanced melanoma were included if they used BRAF/MEK inhibitors or ICIs. The study aimed to indirectly compare ipilimumab/nivolumab and relatlimab/nivolumab, and these combinations versus other first-line treatments, assessing efficacy and safety.	Not given	First-line advanced melanoma treatments: PD-1 + CTLA-4 inhibitors offer best PFS/OS but higher toxicity; PD-1 monotherapy provides favorable efficacy-safety balance. BRAF/MEK inhibitors suit BRAF-mutant patients needing rapid response, though immunotherapy yields better long-term outcomes. Personalized therapy is recommended based on mutation status, disease burden, and toxicity tolerance.	PD-1 monotherapy was the safest option, while PD-1 + CTLA-4 combinations required closer monitoring due to higher toxicity. BRAF/MEK inhibitors had different but predictable AEs, emphasizing the need for tailored management based on treatment choice.	Not given
Migden et al. [31]	Open-label, multicenter, non-randomized study	Expansion cohorts of the Phase 1 study (n = 26) metastatic-disease cohort of the Phase 2 study (N = 59)	Phase 1 study the males 21 (81%) phase 2 study the males was 54 (92%)	Not mentioned	Metastatic cutaneous squamous cell carcinoma	Phase 1: primary endpoint - safety and side-effect profile of cemiplimab. Phase 2: non-randomized, global pivotal trial in advanced cutaneous squamous-cell carcinoma, with primary endpoint - objective response rate by independent central review.	both studies, secondary end points included the duration of response, progression- free survival, overall survival, and toxic effects.	Cemiplimab showed a 47% response rate and 61% durable disease control, with rapid onset (median 1.9 months) and over half lasting >6 months. At cutoff, 82% maintained response; 12-month PFS and OS rates were 53% and 81%. Efficacy was similar in regional (43%) and distant (49%) metastases.	The most common adverse events were diarrhea (occurring in 27% of the patients), fatigue (24%), nausea (17%), constipation (15%), and rash (15%)	None
Hasmat et al. [32]	Non-randomized, prospective trial	19 patient	14 male (74%), 5 female ( 26%)	None	All patients with locally advanced or metastatic CSCC	The primary end point was objective response rate (ORR) described as those with complete (CR) or partial response (PR).	Secondary endpoints included time to response, disease-control rate (CR, PR, or SD), PFS, duration of response, OS, treatment toxicity, and response predictors.	The overall ORR was 68% (13/19) while the DCR was 79% (15/19). CR and PR were noted in 10 (53%) and 3 (16%) cases The median time to observed response was 2.1 months (0.7–3). Progression of disease was noted after CR in two patients with one occurring at 21 weeks post treatment completion and the other at 7 weeks	Toxicity occurred in 10 patients (53%), mostly grade 1 fatigue (60%); one patient had a Grade 3 rash requiring hospitalization for steroids.	0 = 5, 1 = 11, 2=3
Ríos-Viñuela et al. [33]	Prospective observational study	13	12 male and 1 female	None	Six patients (46%) had locally advanced cScc, while 7 (54%) had metastatic cScc.	The main outcome measurement was treatment response.	Secondary objectives were to evaluate treatment-related adverse events (AEs) and progression-free survival (PFS)	Patients received a median of 6 cycles of cemiplimab (range 1-23). Overall,8 cases (62%) responded to cemiplimab after a median of2 cycles (range 2-4). Three patients (23%) showed a complete response and 5 (38%) a PR.	Treatment-related toxi-city, 6 patients (46%) developed AEs. Most of them were mild (G1), and none of the patients in our series presented a serious or lethal adverse reaction	None
Ferris et al. (CheckMate 141) [38]	Randomized, open-label	361 patient	Not reported	Owing to small sample sizes, statistical significance is not reported for the exploratory immune cell biomarker analysis.	Patient with cScc 361 randomized patients, 147 of 240 patients in the nivolumab arm (61.3%) and 74 of 121 in the IC arm (61.2%) had previously received cetuximab . Among patients with prior cetuximab exposure randomized to the IC arm, 41 (55.4%), 32 (43.2%), and 1 (1.4%) received methotrexate, docetaxel, and cetuximab, respectively	The primary endpoint was overall survival (OS)	Secondary endpoints were progression-free survival and objective response rate.	N patients without prior cetuximab, nivolumab significantly improved overall survival (OS: 8.2 vs. 4.9 months; HR 0.52) and 12-month OS (38.5% vs. 11%), with higher objective response rates (17.2% vs. 4.3%) and durable responses. In patients with prior cetuximab, OS benefit was smaller (7.1 vs. 5.1 months; HR 0.84), though nivolumab maintained a better safety profile.	Treatment-related adverse events were less frequent and less severe with nivolumab than with IC	In prior exposure to cetuximab 0 = 41, 1 = 175, 2 = 3 not reporter = 2. In patient without prior exposure to cetuximab 0 = 31, 1 = 108, 2 = 1.
Rischin et al. [34]	Open-label, non-randomized, multicenter, international, Phase 2 study	115 patient	Male 102 (88.7%); female 13 (11.3%)	Tumor mutational burden. Median TMBs were 61.4 and 53.2 mutations per megabase among responding patients in Group 3 and Group 1 and were 13.7 and 19.4 mutations per megabase among non-responding patients in Group 3 and Group 1.	Metastatic cutaneous squamous cell carcinoma	The primary objective for each group was objective response rate (ORR) per independent central review (ICR)	Secondary endpoints included ORR by investigator review (INV), duration of response (DOR) per ICR and INV, and safety and tolerability.	ORR per ICR was 41.1% (95% CI, 28.1% to 55.0%) in Group 3, 49.2% (95% CI, 35.9% to 62.5%) in Group 1, and 45.2% (95% CI, 35.9% to 54.8%) in both groups combined	Overall, the most common adverse events regardless of attribution were fatigue (27.0%) and diarrhea (23.5%).	0 = 48, 1= 67

Across 20 studies, strong evidence supports using ICIs, particularly PD-1/PD-L1 and CTLA-4 blockers, as frontline or salvage therapy for melanoma. These studies consistently demonstrate benefits in ORR, PFS, and OS, while also emphasizing biomarkers and safety, underscoring their short- and long-term effectiveness.

The ORR, which is the proportion of patients with complete or partial tumor shrinkage, was the most reported outcome [32-34,36,38,40]. Early-phase studies showed high ORRs with combination immunotherapies, e.g., nivolumab monotherapy achieved 43% [18], and adoptive T-cell transfer plus dendritic cell vaccination reached 100% [36] - though sample sizes were small and trials were nonrandomized [18,36]. Trends suggest checkpoint inhibitor combinations and targeted therapies have strong potential, especially in first-line. Standardized response criteria are needed [42], and ORR should be interpreted alongside PFS, which measures response durability [43].

Monotherapy and salvage therapies generally yield shorter PFS. For instance, avelumab had a median PFS of 2.7 months in MCC, reflecting aggressive disease [26]. Real-world data in BRAF-mutant metastatic melanoma showed longer PFS with ICIs [17]. Remarkably, TIL plus nivolumab achieved a mean PFS of 615 days in select patients [19]. In Rischin et al., fixed-dose cemiplimab therapy demonstrated the longest PFS in metastatic cSCC, reaching 18 months [34].

Flaherty et al. demonstrated that PFS strongly correlates with OS, supporting its use as a surrogate endpoint to accelerate drug approval [41]. While PFS remains key for evaluating immunotherapy in skin cancers, variability in study design, populations, and endpoint reporting limits its clarity. Future trials should standardize PFS definitions, include control groups, and incorporate biomarker stratification for more reliable results.

OS is the gold-standard endpoint in oncology, reflecting treatment effectiveness while accounting for subsequent therapies and resistance [44]. Immunotherapy, particularly PD-1 and CTLA-4 combination regimens, has markedly improved OS in skin cancers, with some patients achieving over five-year survival [20] . However, cross-study comparisons are limited by heterogeneous populations, variable follow-up, inconsistent controls, and incomplete reporting of hazard ratios or confidence intervals. Data on specific subgroups - older adults, patients with brain metastases, and racial/ethnic minorities - remain scarce. TIL-based studies often could not report median OS due to small cohorts and ongoing responses [19,36].

Treatment effectiveness is primarily assessed via OS, PFS, and objective response rate (ORR), while secondary outcomes-such as safety, quality of life, and predictive biomarkers-provide additional insight [43]. DOR is key, indicating how long patients benefit, for example, Tjulandin et al. reported that prolgolimab achieved responses >6 months with an ORR of 31.7-33.3% [29].

Disease control rate (DCR), which includes complete, partial, and stable responses, is another key measure. Jamal et al. reported a 57% DCR using ipilimumab combined with carboplatin and paclitaxel in advanced melanoma [16], while Greaney et al. showed significant disease control with IL-12 plasmid electroporation [35]. These findings underscore the value of assessing both DCR and ORR to fully capture immunotherapy benefits.

Recent analyses emphasize the importance of time to response (TTR) in immunotherapy, due to delayed responses or pseudo-progression. Olson et al. reported a median TTR of ~2 months with pembrolizumab plus low-dose ipilimumab after PD-1 failure [30], while Kim et al. observed delayed intracranial responses in brain metastasis patients receiving ICI and radiotherapy [39]. These findings highlight the need to consider immune response kinetics when evaluating treatment efficacy. Table 3 summarizes the comparator arms, control groups, and reported confidence intervals from the included studies evaluating immunotherapy in skin cancer.

**Table 3:** Summary of comparator arms, control arms, and confidence intervals in studies on immunotherapy for skin cancer.

Study	Comparator arm(s)	Control arm	Reported confidence interval
Wolchok et al. [20]	Nivolumab and ipilimumab versus nivolumab alone	Ipilimumab	Not reported
Long et al. (NeoTrio Trial) [2]	Pembrolizumab versus sequential versus concurrent immunotherapy	Pembrolizumab	Not reported
da Silveira Nogueira Lima et al. [15]	Programmed cell death protein 1 inhibitors versus BRAF and MEK inhibitors versus others	Chemotherapy	Not reported
Pasquali et al. [28]	Programmed cell death protein 1 inhibitors versus cytotoxic T-lymphocyte-associated protein 4 inhibitors versus chemotherapy	Chemotherapy	Not pooled
Tjulandin et al. [29]	Prolgolimab 1 milligram per kilogram versus 3 milligrams per kilogram	None	Not reported
Hao et al. [27]	Programmed cell death protein 1 and cytotoxic T-lymphocyte-associated protein 4 combination versus chemotherapy or ipilimumab	Chemotherapy or ipilimumab	Not uniformly reported
Kim et al. [39]	Immune checkpoint inhibitor versus immune checkpoint inhibitor with radiotherapy	Immune checkpoint inhibitor monotherapy	Not provided
Khattak et al. (KEYNOTE-716) [22]	Pembrolizumab versus placebo	Placebo	Not discussed
Jamal et al. [16]	Carboplatin and paclitaxel with ipilimumab versus historical outcomes	None	Not reported
L'Orphelinet al. [19]	Tumor-infiltrating lymphocyte with programmed cell death protein 1 versus dacarbazine	Dacarbazine	Not reported
Greaney et al. [35]	None (interleukin-12 electroporation study)	None	Not reported
Lövgren et al. [36]	None (adoptive cell therapy and dendritic cell vaccination)	None	Not reported
Olson et al. [30]	None	None	Not specified
Luke et al. [17]	First-line targeted therapy versus first-line immunotherapy	Varies by cohort	Not clearly reported
Kaufman et al. [26]	None (single-arm avelumab trial)	None	95% confidence interval: 7.5 to not estimable
Ascierto et al. (2019) [18]	Nivolumab versus dacarbazine	Dacarbazine	95% confidence interval: 44.1%-57.9% (nivolumab), 16.1%-27.6% (dacarbazine)
Ascierto et al. (2020) [40]	Ipilimumab 10 milligrams per kilogram versus 3 milligrams per kilogram	Ipilimumab 3 milligrams per kilogram	Not reported
Olivier et al. [21]	None (narrative review)	None	Not applicable
Boutros et al. [14]	Ipilimumab and nivolumab versus relatlimab and nivolumab; programmed death-ligand 1/BRAF/MEK triplet therapy	Chemotherapy (implied)	Response rate risk ratio: 0.99; progression-free survival hazard ratio: 0.56
Flaherty et al. [41]	Progression-free survival and overall survival correlation in dacarbazine-controlled trials	Dacarbazine	Not specified
Chiarion-Sileni et al. [37]	Ipilimumab retreatment (3 mg/kg) in pretreated melanoma patients	None (single-arm EAP)	Not specified
Migden et al. [31]	None (single arm cemiplimab trail)	None	Phase 1 study, a response to cemiplimab was observed in 13 of 26 patients (50%; 95% confidence interval (CI), 30 to 70). In the metastatic-disease cohort of the Phase 2 study, a response was observed in 28 of 59 patients (47%; 95% CI, 34 to 61)
Hasmat et al. [32]	None (single arm cemiplimab trail)	None	Not specified
Ríos-Viñuela et al. [33]	None (single arm cemiplimab trail)	None	Not specified
Ferris et al. (CheckMate 141) [38]	Nivolimab for cSCC with prior cetuximab vs without prior cetuximab	With cetuximab vs without cetuximab	OR (95% CI) 1.69 (0.59-4.80)
Rischin et al. [34]	Cemiplimab fixed dose 350 mg every 3 weeks vs 3m g/kg every 2 weeks	3 mg/kg every 2 weeks	45.2 (35.9 to 54.8)

Few studies have assessed health-related quality of life (HRQoL) as a secondary endpoint. The KEYNOTE-716 trial, using EORTC QLQ-C30 and EQ-5D-5L, showed that adjuvant pembrolizumab did not significantly reduce HRQoL despite long treatment duration [22]. This underscores the importance of including HRQoL in trials, especially as survivorship and long-term outcomes gain focus.

Compared to controls, anti-PD-1 therapy was associated with a significantly lower cumulative incidence of BCC (log-rank test, P < 0.001), while BRAFi treatment showed no significant difference (P = 0.453). When evaluated individually against controls, BRAFi was linked to a significantly higher incidence of cSCC (log-rank test, P < 0.001), while anti-PD-1 therapy showed no significant difference (P = 0.320). This demonstrates overall superiority in using anti-PD-1 therapy in patients of melanoma with concomitant BCC and cSCC [45].

Immunotherapy’s proven survival benefits and new standards of care underscore its expanding role beyond melanoma, offering hope for improved outcomes across multiple malignancies.

Monotherapy vs. combined therapy vs. chemotherapy results

A critical clinical issue is identifying the best approach among chemotherapy, immunotherapy alone, and combined immunotherapy.

Chemotherapy

The comparison with traditional chemotherapy remains clinically relevant, as demonstrated by nivolumab's significant survival advantage over dacarbazine (three-year OS: 51.2% vs. 21.6%). This highlights the substantial improvement of modern immunotherapies and targeted agents over conventional chemotherapeutic approaches in skin cancer [20].

Immunotherapy as monotherapy 

The anti-PD-1 monotherapy demonstrated significant efficacy, with nivolumab achieving a three-year OS rate of 51.2% compared to 21.6% for dacarbazine in BRAF wild-type melanoma [18]. The anti-PD-1 agent prolgolimab maintained efficacy across different dosing schedules (38.1% vs. 28.6% ORR) while demonstrating durable responses [29].

Similarly, avelumab showed promising activity in MCC with a 33% ORR [26]. 

The use of cemiplimab showed a superior survival rate and pathological improvement in comparison to novilumab and to pembrolizumab [31,38,46].

Immunotherapy as combination therapy

Nivolumab plus ipilimumab showed superior median OS (72.1 months) vs. monotherapy (nivolumab: 36.9; ipilimumab: 19.9 months) but higher grade 3/4 toxicity (59%) [14,20]. BRAF/MEK inhibitors (encorafenib/binimetinib) achieved a median OS of 33.6 months in BRAF-mutant melanoma [40].

Novel immunotherapy

ICIs targeting PD-1 and CTLA-4 have established themselves as standard treatments. However, many patients exhibit primary resistance or develop immune escape after a positive response. This has prompted investigations into novel immunotherapeutic strategies designed to boost immune activation, overcome resistance, and enhance clinical outcomes.

Recent advances in cutaneous tumor immunotherapy include intertumoral IL-12 gene therapy [45-47]. Unlike systemic approaches with high toxicity, local delivery via plasmid or mRNA IL-12 (“in situ vaccination”) elicits strong local and systemic immune responses while minimizing adverse effects [35,48]. Plasmid IL-12 electroporation increases intertumoral CD3⁺/CD8⁺ T cells and circulating antigen-specific IFN-γ, supporting its potential in combination with other immunotherapies like checkpoint inhibitors [35,48].

Other immunotherapy options examined in this review include checkpoint inhibitors. Higher dose ipilimumab (10 mg/kg) improved median OS (15.7 months) versus 3 mg/kg (11.5 months) but increased toxicity after 61 months of follow-up [40]. A phase 3 RCT with ≥6.5 years follow-up showed nivolumab plus ipilimumab significantly improved five-year OS, supporting its use as first-line therapy for advanced melanoma [20].

A phase I/II trial by L'Orphelin et al. combining nivolumab with autologous TILs in metastatic melanoma showed a 75% ORR, including three prolonged complete responses, highlighting the synergy of PD-1 blockade with personalized cell therapy [19] .

Lövgren et al. piloted a combination of TIL therapy with tumor-lysate-loaded dendritic cells in four advanced melanoma patients, achieving clinical responses in all, highlighting the potential of synergistic immune-stimulatory strategies [36].

Tjulandin et al. introduced prolgolimab, an IgG1 monoclonal antibody that targets PD-1. In the MIRACULUM phase II trial, prolgolimab showed an ORR of 32% across two dosing regimens and a favorable safety profile. Although it operates similarly to other PD-1 inhibitors, its development adds to the variety of global checkpoint blockade options available [29].

Olson et al. investigated the effectiveness of low-dose combination therapy using pembrolizumab (PD-1 inhibitor) with ipilimumab (CTLA-4 inhibitor) in patients who had not responded to PD-1/PD-L1 treatments. This phase II single-arm study reported an ORR of 29%, indicating a significant response rate in a population resistant to prior therapies. The use of low-dose ipilimumab aims to minimize toxicity while enhancing T-cell function. These results suggest a potential salvage strategy for checkpoint-refractory melanoma and support the logical reintroduction of CTLA-4 inhibition [30].

Innovative immunotherapies, beyond checkpoint inhibitors, such as adoptive cell therapy, intertumoral cytokine activation, and enhanced antigen presentation, show promising responses in skin cancer. These approaches signal a move toward more personalized, effective treatments, with future studies needed to optimize integration with standard care and incorporate predictive biomarkers. Table 4 provides a structured overview of key clinical studies evaluating immunotherapy in skin cancer. It summarizes the type of intervention (monotherapy or combination therapy), therapies used, and the clinical outcomes reported across these trials.

**Table 4:** Summary of key clinical studies evaluating immunotherapy interventions in skin cancer. It categorizes the interventions by type (monotherapy vs. combination therapy), lists the associated studies, specifies the immunotherapeutic agents used, and highlights the primary clinical outcomes such as overall survival (OS), progression-free survival (PFS), and objective response rates (ORR). The findings provide comparative insights into the efficacy and potential of various immunotherapeutic strategies in treating skin cancers.

Type of intervention	Studies involved	Type of therapy	Result
Immunotherapy as monotherapy	Ascierto et al., 2019 [18]	Nivolumab	The 3-year OS rate was more than double nivolumab 51.2% vs dacarbazine 21.6% in melanoma
	Ascierto et al., 2020 [40]	Ipilimumab	In advanced melanoma the higher dose of Ipilimumab (10 mg/kg) has better OS the lower dose of (3 mg/kg ) median OS: 15.7 vs. 11.5 months in melanoma
	Tjulandin et al. [29]	Prolgolimab	Prolgolimab maintained efficacy across different dosing schedules (38.1% vs. 28.6% ORR) while demonstrating durable responses in melaoma
	Greaney et al. [35]	Intratumoral delivery of IL-12 through tavokinogene telseplasmid electroporation (tavo)	Indicated that local treatment with tavo can induce a systemic T-cell response and recruit T cells to the tumor microenvironment.
	Kaufman et al. [26]	Avelumab	The study found an objective response rate in metastatic Merkel cell carcinoma of 31.8%, with 9.1% achieving complete responses and 22.7% partial responses in melanoma
	Migden et al. [31]	Cemiplimab	Cemiplimab showed a 47% response rate and 61% durable disease control, with rapid onset (median 1.9 months in cSCC
	Hasmat et al. [32]	Cemiplimab	The overall ORR was 68% (13/19) while the DCR was 79% (15/19). CR and PR were noted in 10 (53%) and 3 (16%) in cSCC
	Ríos-Viñuela et al. [33]	Cemiplimab	Three patients (23%) showed a complete response and 5 (38%) a PR in cSCC
	Ferris et al. (CheckMate 141) [38]	Nivolumab	In cSCC patients without prior cetuximab, nivolumab significantly improved overall survival (OS: 8.2 vs. 4.9 months;
	Rischin et al. [34]	Cemiplimab	In cSCC fixed dose of cemiplimab showed ORR per ICR was 41.1% (95% CI, 28.1% to 55.0%)
Immunotherapy combination as Immunotherapy- Immunotherapy	L'Orphelin et al. [19]	Nivolumab (anti-PD-1) + tumor-infiltrating lymphocytes (TILs)	Sustained complete responses were observed in three patients beyond the 12-month study period, with a mean progression-free survival of 615 days. The trial
	Olson et al. [30]	Pembrolizumab (anti-PD-1) + low-dose ipilimumab (anti-CTLA-4).	The median progression-free survival was 5.0 months, and the median overall survival was 24.7 month
	Boutros et al. [14]	Combination comparisons: ipilimumab + nivolumab (dual checkpoint blockade). Relatlimab + nivolumab (LAG-3 + PD-1 inhibition).	Relatlimab/nivolumab showed comparable efficacy to ipilimumab/nivolumab (PFS HR 0.99, ORR RR 0.99) with a distinct safety profile
	Hao et al. [27]	Nivolumab + ipilimumab.	Anti-PD1 monotherapy and nivolumab plus ipilimumab, improved ORR and prolonged PFS of patients with advanced melanoma
	Lövgren et al. [36]	Tumor-infiltrating lymphocyte (TIL) adoptive cell therapy (ACT) combined with dendritic cell (DC) vaccination	Combination of TIL ACT and tumor lysate-loaded DC vaccination is feasible and safe and we observed impressive clinical responses in all patients treated with the combination
Combination therapy as immunotherapy-targeted therapy	Long et al. [2]	3 groups: 1st group pembrolizumab only, 2nd sequential therapy (1 week of dabrafenib plus trametinib followed by pembrolizumab; 3rd concurrent triple therapy (pembrolizumab with dabrafenib plus trametinib	Pathological response was observed in 11 of 20 patients receiving pembrolizumab alone, 10 of 20 in the sequential group, and 16 of 20 in the concurrent group; complete responses occurred in 6, 3, and 10 patients respectively Radiographic responses were seen in 6 of 20 patients with pembrolizumab alone, 10 of 20 in the sequential group, and 14 of 20 in the concurrent therapy group
	Pasquali et al. [28]	Combination comparisons: BRAF + MEK inhibitors (e.g., dabrafenib + trametinib). Anti-CTLA4 + anti-PD1 (ipilimumab + nivolumab).	BRAF inhibitors are effective only in people with BRAF‐mutated melanoma; BRAF inhibitors combined with MEK inhibitors are the most effective regimen in people with BRAF‐mutated melanoma (at least in terms of progression‐free survival); and anti‐PD1 monoclonal antibodies are the least toxic regimen, but the combination of immune checkpoint inhibitors has highest toxicity.
	Boutros et al. [14]	PD-(L)1 inhibitors + BRAF/MEK inhibitors (e.g., pembrolizumab + dabrafenib + trametinib).	PFS: PD-(L)1/BRAF/MEK triplets outperformed ipilimumab/nivolumab (HR 0.56, 95% CI 0.37–0.84) with a 99% probability of being the best treatment (SUCRA: 100%) ORR: Triplets (PD-(L)1/BRAF/MEK) and BRAF/MEK combinations had higher ORR vs. ipilimumab/nivolumab (RR 3.07 and 2.99, respectively).
Combination therapy: immunotherapy-radiotherapy	Kim et al. [39]	(Nivolumab/ipilimumab) + radiotherapy (SRS/WBRT) in brain metastasis	Immunotherapy combination therapy or immunotherapy combined with radiotherapy showed better local efficacy than immunotherapy monotherapy for treating melanoma brain metastasis.
Combination immunotherapy-chemotherapy	Jamal et al. [16]	Combination therapy: ipilimumab (anti-CTLA-4) + carboplatin/paclitaxel (chemotherapy)	The treatment demonstrated anti-tumor activity but did not significantly improve overall survival (OS) compared to existing therapies.

Special population

In advanced melanoma with brain metastases, combination immunotherapy (nivolumab + ipilimumab) achieved the highest ORR (57%), followed by ICI plus radiotherapy (42%) and ICI monotherapy (15%), indicating superior intracranial activity with combination regimens. Grades 3-4 adverse events occurred in 4% with ICI-radiotherapy, and CNS toxicities in 8%, suggesting a favorable efficacy-to-toxicity balance for selected patients [39].

Schmidberger et al. reported that ipilimumab after radiotherapy (SRS or WBRT) was linked to improved OS, 11 months compared to just three months, in those who received ipilimumab before radiation [48].

Biomarkers and their significance

Biomarker assessment is increasingly crucial for guiding immunotherapy in skin cancers. Studies in advanced melanoma, cSCC, and MCC highlighted tumor-specific mutations, immune checkpoint expression, and circulating immune markers as predictors of response. BRAF status, especially V600E, was frequently used for patient selection or outcome stratification [14,15,17,18,29,40]. For example, Jamal et al. found variable responses based on BRAF/NRAS mutations and inflammatory markers (CCL4 and CXCL8) [16], while Long et al. and Tjulandin et al. confirmed universal BRAF V600 positivity in their cohorts [2,39]. Conversely, Ascierto et al. and Wolchok et al. enrolled only BRAF wild-type patients to standardize nivolumab efficacy analyses [18,20].

PD-L1 is frequently studied as a biomarker, but its predictive value is inconsistent [14-16,29,35]. Meta-analyses by da Silveira Nogueira Lima et al. and Tjulandin et al. indicated PD-L1 alone poorly predicts response to PD-1 blockade [15,29]. However, Kim et al. showed that combining PD-L1 status with BRAF mutations identified melanoma brain metastasis subgroups with better outcomes under combination therapy [39]. Similarly, Olivier et al. found that high TMB or microsatellite instability (MSI) predicted prolonged benefit from nivolumab plus ipilimumab in the CheckMate 067 trial [21].

Interestingly, some studies have investigated circulating and functional immune biomarkers beyond tumor genomics. Greaney et al. found that higher baseline CD39⁺ CD8⁺ T cells predicted better response to immune checkpoint blockade, correlating with longer PFS. Responders also showed increased Ki-67⁺ proliferating T cells post-treatment [35]. Similarly, Lövgren et al. observed that TILs combined with dendritic cell vaccines led to expansion and lasting presence of NY-ESO-1-specific T cells, changes in TCR clonality, and evolving PD-1 expression, especially in patients achieving complete responses [36].

Many trials highlighted BRAF status as a key biomarker for treatment selection and stratification. PD-L1 was frequently assessed but showed inconsistent predictive value, suggesting it alone is insufficient. Increasingly, studies are incorporating immune cell profiling and circulating biomarkers to enable non-invasive monitoring of treatment response.

Not all studies included biomarker evaluations. For example, Kaufman et al. examined avelumab in patients with chemotherapy-refractory MCC. They reported the survival and response outcomes but did not comment on molecular predictors [26]. Similarly, the expanded access initiative led by Chiarion-Sileni et al. [37] and an early-phase trial by L'Orphelin et al. [19] did not include formal tests to measure specific biological markers, focusing instead on safety, feasibility, and patient responses to treatments. The same thing in cSCC, there was a lack of biomarkers in all studies that were included [31-33,38]. This lack of information about biological markers highlights a gap in understanding which patient groups benefited the most from the treatments. It also demonstrates the need for future research that includes these crucial markers to understand their effects better.

BRAF status is a key biomarker for therapy selection, while PD-L1 shows inconsistent predictive value. Immune cell profiling and circulating biomarkers are increasingly used for non-invasive response monitoring. Table 5 provides a consolidated overview of biomarkers evaluated across studies, highlighting their prognostic and predictive significance in immunotherapy response for skin cancers.

**Table 5:** Summary of biomarkers and their significance.

Biomarker(s)	Author	Prognostic/predictive association
BRAF mutation V600EK, wild-type	Ascierto et al., 2019 [18]	BRAF mutations are linked to favorable response to BRAF-MEK inhibitors therapy. Tumors without BRAF mutation (wild type), association with better outcomes with PD-1 blockade.
PD-L1 expression	Hao et al. [27]	High expression is correlated with adverse outcomes in different tumors as malignant melanoma, and better response to ICI. However, more studies are needed.
TMB (Tumor mutation Burden)	Olivier et al. [21]; Rischin et al. [32]	High TMB is linked to improved response to immune checkpoint blockade, likely due to a higher neoantigen load. Tumor mutational burden Median TMBs were 61.4 and 53.2 mutations per megabase among responding patients
MSI (microsatellite instability)	Olivier et al. [21]	Although rare in melanoma, the presence of MSI may predict better response to immunotherapy.
LDH (lactate dehidrogenase)	Ascierto et al., 2020 [40]	Elevated LDH is a negative prognostic indicator, associated with worse overall survival.
CD39+ CD8 T cells	Greaney et al. [35]	Higher baseline levels of CD39+ CD8+ T cells, are linked to improved clinical response and prolonged PFS. CD8+ T cell markers are linked to tumor reactivity.
Circulating and intratumoral T cells, T cell repertoire.	Greaney et al. [35]	Clinical benefit was associated with the persistence of tumor-infiltrating lymphocytes TCR clones. Increased clonality and overlap of TCR clones in blood linked with clinical response. Responders had higher intratumoral CD3+ T cells.
TCR clonality CD4/CD8 ratio in TILs, DC maturation marker	Lövgren et al. [36]	Persistence of TIL-derived TCR clones in blood was associated with clinical response. CD8+ phenotype was common in responders.
Peripheral and local immune signatures	Jamal et al. [16]	Patients who responded to treatment showed higher levels of intratumoral CD3+ cells, while non-responders exhibited elevated systemic levels of CXCL8, CCL4, and CD8+/PD-1+ T cells. These patterns suggest predictive value for immunotherapy outcomes.

Adverse effects reported across studies

irAEs of varying severity can affect any organ system and continue to present a substantial clinical challenge as immunotherapy expands into earlier disease scenarios and a broader range of patients. Low-grade irAEs observed across the selected studies were generally manageable and included diarrhea, rash, pruritus, vitiligo, myositis, and arthritis. Dermatological findings were often associated with anti-PD-1/PD-L1 therapies [31-34,38,49]. High-grade irAEs require adjustments to immunotherapy, as well as the use of corticosteroids or other immunosuppressants. Common toxicities included endocrinopathies such as thyroid dysfunction, adrenal insufficiency, and hypophysitis. Gastrointestinal involvement, including severe colitis, diarrhea, and hepatitis, was frequently observed with CTLA-4 blockade and combined ICIS [16,49]. Immune-related pneumonitis is rarely seen; however, it carries a high mortality risk, especially in older individuals and those with underlying pulmonary pathology [20]. While uncommon, severe neurologic adverse events like Guillain-Barré syndrome and encephalitis have been documented in specific instances [16,39]. Emerging treatments such as intratumoral IL-12 plasmid electroporation may cause localized adverse events, such as inflammation at the injection site. Preliminary studies suggest that intratumoral IL-12 may reduce systemic toxicity compared to ICIs, but this requires validation in larger controlled trials. Preexisting autoimmune conditions can increase the risk of irAEs [35]. The remaining challenge for future trials is identifying reliable irAE prediction biomarkers, as current links to gut microbiota or cytokine profiles remain indeterminate [2] . A further limitation across many studies is a lack of comprehensive long-term data on immune-related toxicity. Future trials should also address the gap between reporting and the real-world incidence of irAEs.

Limitation

We recognize that this study has some limitations: the variations in study designs, such as different patient populations, follow-up times, and outcome measures, make comparisons of trial results challenging, mainly when some endpoint trials focus on OS while others emphasize PFS. Combination treatments like nivolumab plus ipilimumab show positive long-term outcomes, but data on toxicity and long-term efficacy beyond six years of follow-up are still needed [20]. Most studies are limited to small samples, case reports, or even lack a comparator arm [14] . Therefore, assessing functional status or identifying superior treatments is challenging. Most studies utilize endpoints like ORR, commonly accepted in immunotherapy trials, but may not always correlate with long-term benefits. English was the only language used in this research, which might have resulted in missing other studies published in different languages. Manual data entries also tend to introduce transcription errors.

Furthermore, many crucial prognostic factors-such as LDH levels, TMB, and detailed baseline characteristics - are often incomplete [17]. Measurement of progression adds further complexity, as some studies assess it using RECIST 1.1 while others rely on immune-related response criteria; whether patients receive therapies after progression further complicates comparisons. No study has compared intertumoral IL-12 therapies with any established ICIs, nor has a direct collective comparison between various immunotherapy approaches, particularly between monotherapy, intertumoral, and combination regimens.

Future direction and scope

For future studies, researchers should focus on conducting large, randomized trials comparing immunotherapy monotherapy with combination regimens and long-term follow-up beyond five years to better evaluate the response and late-onset side effects. They should also report irAEs, including incidents and severity, over an extended period to assess the safety profile of these therapies.

Using standardized primary endpoints such as RECIST1.1 and immune-related response criteria would enable better cross-trial comparisons and meta-analyses. Integrating comprehensive biomarkers like PD-L1 expression, tumor mutational burden, and lactate dehydrogenases (LDH) can refine patient selection and personalize treatment strategies. 

Documenting post-progression is essential for interpreting survival outcomes and the sequence of treatment. Combining systemic checkpoint inhibitors with localized therapy, such as intertumoral IL-12, would deliver local and systemic immune responses while minimizing overall toxicity.

## Conclusions

In conclusion, our systematic review demonstrates that combination immunotherapy regimens offer superior efficacy compared to monotherapy across a range of skin cancers, including melanoma, MCC, and non-melanocytic skin cancers. These findings underscore the necessity of careful patient selection, considering tumor subtype, stage, and comorbidities, to maximize the benefit and minimize the adverse effects. and long-term safety monitoring remain essential to optimizing clinical outcomes. Early-phase trials of intratumoral IL-12 show promising results, eliciting local and systemic immune responses with fewer side effects, suggesting a valuable role in multimodal treatment regimens.
